# Automation of Living Clinical Practice Guideline Development: Scoping Review

**DOI:** 10.2196/103273

**Published:** 2026-10-28

**Authors:** Abdullah Altammami, Olga Ares-Rufo, Anas Alzahrani, Abdullah Alkhotani, Dinesh Mital, Jose F Florez-Arango

**Affiliations:** 1 Health Informatics Department College of Public Health and Health Informatics King Saud Bin Abdulaziz University for Health Sciences Riyadh Saudi Arabia; 2 Department of Health Informatics Rutgers, The State University of New Jersey New Brunswick, NJ United States; 3 Center for Biomedical Informatics and Biostatistics (CB2) University of Arizona Tucson, AZ United States; 4 Department of Preventive Medicine and Public Health Faculty of Medicine King Abdulaziz University Jeddah Saudi Arabia; 5 King Abdulaziz University Hospital King Abdulaziz University Jeddah Saudi Arabia

**Keywords:** clinical practice guidelines, automation, artificial intelligence, machine learning, natural language processing, computer-interpretable guidelines, GRADE, AGREE II, evidence-based medicine, decision support systems, living guideline, Grading of Recommendations Assessment, Development and Evaluation, Appraisal of Guidelines for Research and Evaluation II

## Abstract

**Background:**

Clinical practice guidelines (CPGs) translate research evidence into actionable recommendations, but their development faces mounting challenges. With dozens of trials and systematic reviews published daily, the traditional 18- to 24-month development cycle struggles to incorporate emerging evidence, and manual screening, synthesis, and quality assessment consume thousands of person-hours per guideline. The resulting knowledge translation pipeline is slow, inconsistent, and quickly outdated.

**Objective:**

This review aimed to chart the computational methods empirically tested for automating CPG development, the tasks they address, and their evaluation measures, and to map where that evidence is concentrated and where it is absent for living guidelines.

**Methods:**

The review followed the Joanna Briggs Institute scoping review framework and PRISMA-ScR (Preferred Reporting Items for Systematic Reviews and Meta-Analyses extension for Scoping Reviews) guidance. We searched PubMed and Scopus for studies published between January 2001 and December 2025, supplemented by backward and forward citation searching. Eligible studies used automated methods for a guideline development task, reported original research with empirical testing, and gave sufficient methodological detail. Two reviewers independently screened records and extracted data. Findings were charted across 4 pillars: retrieval, extraction, encoding, and evaluation.

**Results:**

A total of 47 studies met eligibility criteria, spanning 2001 to 2025, with a marked increase after 2015 and a surge in large language model (LLM) work in 2024-2025. Pillar counts (nonexclusive) were as follows: retrieval, 6; extraction, 15; encoding, 26; and evaluation, 17. In retrieval pillar, query expansion achieved 80% recall, while semantic search tools traded recall for specificity (98%). In extraction, rule-based natural language processing achieved an F-measure of 81.96% for structured clinical actions and 71.75% for patient states, and hybrid ontology methods covered 86% of eligibility criteria; LLMs performed well on structured fields but degraded on interpretive content. In encoding, approaches progressed from formalisms such as Guideline Interchange Format version 3 (GLIF3) and guideline elements model toward Fast Healthcare Interoperability Resources (FHIR)–based representation; semiautomated tools assisted guideline encoding, and logic verification detected inconsistencies and coverage gaps missed by human reviewers. In evaluation, LLM appraisers assigned higher Appraisal of Guidelines for Research and Evaluation II (AGREE II) scores than human appraisers, by a mean difference of 12.5% in one study (95% limits of agreement –30.6% to 55.5%; intraclass correlation coefficient 0.753) and by 22 to 60 percentage points across domains in another.

**Conclusions:**

Automation shows promise for bounded, well-structured guideline development tasks, while robust evidence for end-to-end, production-scale living guideline pipelines remains limited. No included study demonstrated a validated pipeline connecting all 4 pillars in an operating guideline program, and evidence on clinical safety, workflow integration, and cost-effectiveness is largely absent. Realizing living guidelines will require interoperability standards such as FHIR, and evaluation in real guideline production rather than retrospective benchmarks.

## Introduction

Clinical practice guidelines (CPGs) translate research into actionable recommendations but face mounting challenges. Exponential literature growth—dozens of trials and systematic reviews published daily—creates information overload, making current, comprehensive guidelines increasingly difficult to maintain. Traditional manual processes are resource-intensive, time-consuming, and inconsistent across organizations [1].

Beyond initial development, translating narrative recommendations into clinical decision support systems requires manual encoding by knowledge engineers, a months-long process prone to errors. Quality assessment using AGREE II (Appraisal of Guidelines for Research and Evaluation II) and GRADE (Grading of Recommendations Assessment, Development and Evaluation) instruments is subjective and labor-intensive [2,3]. Keeping guidelines updated remains aspirational rather than a reality.

These challenges create a “knowledge pipeline” from evidence to practice that is slow, inconsistent, and rapidly outdated. Recent advances in AI, natural language processing (NLP), and knowledge representation offer promising solutions [4]. However, the landscape remains fragmented, with studies addressing isolated components using diverse methods, making it difficult to assess the state of the art.

Several reviews have examined subsets of this problem. Earlier work focused primarily on the encoding of guidelines into computer-interpretable formats, cataloging formalisms such as Asbru, Guideline Interchange Format (GLIF), and PROforma without evaluating their performance or scalability [5,6].

More recent reviews have addressed downstream applications—such as the methodology of living guidelines [7] or the integration of encoded guidelines into clinical decision support systems [8]—but none has examined the upstream process by which guidelines are extracted, encoded, and appraised in the first place. To our knowledge, no existing review maps all 4 stages of that process within a unified framework, nor covers the period of rapid large language model (LLM) adoption through 2025. This scoping review therefore aims to determine what kinds of evidence are available on the automation of CPG development, and to chart that evidence across 4 stages: retrieval, extraction, encoding, and evaluation. Specifically, we ask: (1) what computational methods have been empirically tested for automating each stage; (2) what tasks, data sources, comparators, and outcome measures were used to evaluate them; (3) what degree of validation and human oversight each approach required; and (4) where evidence is concentrated or absent with respect to continuously updated, living guidelines.

## Methods

### Overview

Following the Joanna Briggs Institute (JBI) framework for scoping reviews [9], and reporting in accordance with the PRISMA-ScR (Preferred Reporting Items for Systematic Reviews and Meta-Analyses extension for Scoping Reviews; Multimedia Appendix 1) [10], this review maps the available evidence on computational methods for automating CPG development across four pillars: (1) retrieval of relevant literature, (2) extraction of structured information from guideline documents, (3) encoding of guidelines into machine-interpretable formats, and (4) automated quality evaluation. The primary objective was to identify and chart the range of computational methods that have been empirically tested for automating guideline development, the tasks they address, and the outcome measures used to evaluate them, and to map the distribution of that evidence across the 4 pillars in order to expose knowledge gaps and areas of fragmentation. Consistent with scoping review methodology, the review charts what evidence exists and how it has been evaluated; it does not pool results, compare methods against one another, or critically appraise the quality of individual studies [9]. An OSF project for this review was created on March 16, 2026 [11]. The review was not prospectively registered. The project record described the review’s scope but did not specify databases, search strategies, or eligibility criteria in advance, and a registration documenting the methods as conducted was completed retrospectively in August 2026 [12]. The scope of the review evolved during conduct: it was initially framed around 3 pillars, and retrieval was subsequently added as a fourth.

### Search Strategy and Eligibility

We searched PubMed and Scopus on August 10, 2026, for studies published between January 1, 2001, and December 31, 2025. The start date reflects the emergence of shareable computer-interpretable guideline (CIG) formalisms [13].

The search combined 2 sets of terms with OR. The first comprised phrases naming guideline formalisms, appraisal instruments, and automation tasks (eg, “computer-interpretable guideline,” “GLIF3,” “APPRAISE-RS,” and “AGREE II scores”). The second comprised paired terms, each combining a guideline concept with an automation, computational, or process concept (eg, “large language model” AND “clinical practice guideline”; “information extraction” AND “clinical guideline”). Terms were drawn from the vocabulary of studies known to the review team and refined iteratively; the search was not peer-reviewed. Full strategies for both databases are given in Multimedia Appendix 1.

Reference lists of included studies were screened and forward citation searching performed, identifying 8 further records. Gray literature, preprints, and dissertations were not searched. Records were imported into Covidence (Future Evidence Foundation), where 898 duplicates were removed before screening.

### Inclusion Criteria

Studies were eligible if the automated component acted on a guideline development task as defined below, reported original research with empirical testing, and provided sufficient methodological detail for assessment.

### Exclusion Criteria

Studies were excluded if they addressed guideline implementation or adherence only, were opinion pieces without primary data, or were not published in English.

### Operational Definitions and Pillar Assignment

For the purposes of this review, CPG development refers to the processes by which a guideline is produced or updated: identification and screening of evidence, extraction and structuring of evidence and recommendation content, representation of recommendation logic in a machine-interpretable form, and appraisal of the resulting evidence or recommendations. It excludes processes downstream of a completed guideline, namely dissemination, point-of-care execution of an existing guideline in a decision support system, and measurement of clinician adherence. A study was eligible only if the automated component acted on a guideline development task as defined above. Where a computable artifact was authored as part of guideline production, it was treated as development (encoding); where an existing guideline was executed against patient data at the point of care, it was treated as implementation and excluded. A living guideline was defined as a guideline maintained through continuous or scheduled surveillance of new evidence with incremental updating of affected recommendations, rather than through periodic full revision. Studies were assigned to a pillar according to the task the automated component performed: retrieval, where the component identified or screened candidate evidence; extraction, where it parsed narrative text into structured elements; encoding, where it produced or verified a formal, machine-interpretable representation; and evaluation, where it scored or appraised evidence or recommendation quality against an instrument such as GRADE or AGREE II. Studies whose components spanned more than one task were assigned to every applicable pillar, so pillar counts are not mutually exclusive.

### Data Extraction and Synthesis

Titles and abstracts were screened independently and in duplicate by 2 reviewers in Covidence systematic review software, followed by independent duplicate full-text screening against the same criteria. Before formal screening, both reviewers piloted the eligibility criteria on a sample of records and met to resolve interpretive differences; formal screening proceeded once agreement was judged acceptable. Disagreements at either stage were resolved by discussion, with a third reviewer available for arbitration where consensus could not be reached. Records retained after full-text screening were assessed against the operational definitions below. Two reports were excluded at this stage: one was a review and analysis of evaluation criteria rather than original research with empirical testing [14], and one met all other criteria but was published in Spanish [15].

- Pillar 1: Retrieval encompasses computational methods for identifying and retrieving relevant evidence from published literature, including automated search and screening tools [16-18].
- Pillar 2: Extraction encompasses computational methods for parsing narrative guideline text into structured data, including rule-based NLP, ontology-driven approaches, and LLM-based extraction systems [19].
- Pillar 3: Knowledge encoding refers to translating narrative clinical guidelines into formal, machine-interpretable representations that enable automated reasoning and decision support [13,20]. This encompasses representation formalisms (GLIF, guideline elements model [GEM], Arden Syntax, and PROForma) and semiautomated authoring tools. This pillar transforms guidelines from passive documents into active computational artifacts capable of logical verification [21,22].
- Pillar 4: Automated evaluation involves computational approaches to evaluating guideline quality and evidence strength using standardized instruments such as GRADE and AGREE II [23-26].

### Ethical Considerations

This scoping review synthesized data from previously published, publicly available studies. It involved no human participants or animals and no access to individual-level or identifiable patient data. Institutional review board approval and informed consent were therefore not required, consistent with the policies of King Abdulaziz University. No primary data were collected. All included studies are cited in full, and no reproduction of copyrighted material beyond fair academic use was undertaken.

## Results

### Study Selection

The search identified 2577 records: 998 from PubMed, 1571 from Scopus, and 8 through backward and forward citation searching. After removal of 898 duplicate records, 1679 proceeded to title and abstract screening. Two reviewers independently assessed records against the predefined eligibility criteria, with disagreements resolved as described in the “Methods” section. Of these, 1450 records were excluded.

The remaining 229 records were sought for full-text retrieval; 25 could not be obtained, leaving 204 reports for full-text eligibility assessment. Full-text exclusions totaled 157: 61 addressed evidence synthesis or not targeting CPG development automation (R1); 41 focused on clinical decision support or point-of-care use without automating any guideline development process (R2); 29 lacked original research on CPG automation and were reviews, opinions, commentaries, or protocols (R3); 20 did not present a transferable computational method for CPG development, including disease-specific tools or essentially manual approaches (R4); 5 provided insufficient methodological detail to assess the computational approach (R5); and 1 met all other criteria but was not published in English (R6).

The final corpus of 47 studies [17-63] spans 2001-2025 and is characterized in Table 1 and detailed study by study in Table 2. Performance metrics by automation task are summarized in Table 3. The selection process is summarized in Figure 1. Publications showed a marked increase after 2015, with a specific surge in LLM-related work during 2024-2025. The relatively high attrition from 2577 records to 47 inclusions reflects the breadth of the search strategy alongside the specificity of the scope: studies empirically testing computational methods for automating the guideline development process itself—as opposed to using or implementing existing guidelines—represent a considerably smaller subset of the biomedical literature.

**Table 1:** Summary statistics of included studies (N=47 unique studies).

Characteristic		Studies, n (%)
**Automation pillar^a^**		
	Retrieval	6 (13)
	Extraction	15 (32)
	Knowledge encoding	26 (55)
	Automated evaluation	17 (36)
**Transparency**		
	Code or algorithm shared	12 (26)
	Interrater reliability reported	18 (38)

^a^Pillar counts are nonexclusive: 47 unique studies contributed 64 pillar assignments, as several studies addressed more than one pillar.

**Table 2:** Characteristics of included studies categorized by pillar and approach type.

Study	Year	Country	Domain	Retrieval (n=6)	Extraction (n=15)	Encoding (n=26)	Evaluation (n=17)	Approach type
Duftschmid and Miksch [21]	2001	Austria	Neonatology			✓		Logic verification
Duftschmid et al [28]	2002	Austria	Neonatology			✓		Temporal reasoning
Georg et al [29]	2003	France	Hypertension			✓		GEM^a^ encoding
Peleg et al [27]	2003	United States	Cross-domain			✓		CIG^b^ formalism comparison
Boxwala et al [30]	2004	United States	Cross-domain			✓		GLIF3^c^ format
Shiffman et al [31]	2005	United States	Cross-domain			✓	✓	GLIA^d^ instrument
Anselma et al [32]	2006	Italy	Cross-domain			✓		Temporal formalism
Hagerty et al [33]	2006	United States	Hypertension		✓	✓		ML^e^ classifier
Kaiser et al [18]	2007	Austria	General		✓			NLP^f^ (LASSIE^g^)
Hrabak et al [34]	2007	United States	Immunization domain			✓		Terminology standards
Serban et al [35]	2007	Netherlands and Spain	Oncology		✓			NLP pattern extraction
Peleg et al [36]	2008	Israel	Cross-domain			✓		CIG-EMR^h^ mapping (KDOM^i^)
Fiszman et al [37]	2008	United States	cardiovascular	✓	✓			Semantic (SemRep)
Bouaud et al [22]	2009	France	Hypertension			✓		Completeness check
Kaiser and Miksch [38]	2009	Austria	General		✓	✓		Living Guidelines (DELT/A^j^)
Peleg and Garber [39]	2010	Israel	Thyroid			✓	✓	Extended GLIA
De Backere et al [40]	2012	Belgium	ICU^k^			✓		CIG execution
Slaughter et al [41]	2013	United States	Cross-domain			✓		Ontology (C2PO^l^)
Zhu et al [19]	2013	China	Cross-domain		✓			Rule-based NLP
Farkash et al [42]	2013	Israel	Hypertension			✓		Model-driven engineering
Minard and Kaiser [43]	2013	Austria	Cross-domain		✓	✓	✓	ML action classification
Wenzina and Kaiser [63]	2013	Austria	Cross-domain		✓	✓	✓	Heuristic IE^m^ + UMLS^n^ semantic patterns
Wenzina and Kaiser [44]	2014	Austria	Cross-domain			✓		TimeML modeling
Galopin et al [45]	2014	France	Hypertension				✓	Ontology evaluation
Weng et al [46]	2014	United States	Cross-domain		✓			Hybrid NLP and ontology
Bui et al [17]	2015	United States	Multidomain	✓	✓			Query expansion
Stewart et al [24]	2015	United Kingdom	Multidomain				✓	Bayesian network
Llewellyn et al [47]	2015	United Kingdom	Multidomain				✓	Bayesian network
Hu et al [48]	2016	Netherlands	Cross-domain (breast cancer)	✓			✓	Semantic distance (topic-centric)
González-Ferrer et al [49]	2017	Spain	Hyponatremia			✓		PROForma encoder
Afzal et al [50]	2019	South Korea	Multidomain				✓	Context-aware GRADE^o^
Schlegel et al [51]	2019	United States	Cross-domain (diabetes)		✓	✓		NLU^p^ (clinical tractor)
Iglesias et al [20]	2020	Spain	Infectious			✓		Formalism comparison
Fux et al [52]	2020	Israel	Asthma			✓		Multilayer PROForma
Shivers et al [53]	2021	United States	Family planning and STI^q^			✓		FHIR^r^ encoding (WHO^s^ DAK^t^)
Borchert et al [54]	2021	Germany	Oncology	✓	✓		✓	NLP disagreement detection
Šuster et al [25]	2023	Australia	Multidomain		✓		✓	Transformer ML
Lin et al [55]	2023	China	Musculoskeletal	✓				Deep learning screening
Lichtner et al [56]	2023	Germany	Infectious disease			✓		FHIR CPG-on-EBM^u^
López et al [57]	2023	Spain	Psychiatry (ADHD^v^)				✓	GRADE Automation (APPRAISE-RS)
Wan et al [26]	2025	China	Surgery				✓	LLM^w^ (AGREE II^x^ and RIGHT^y^)
Dos Santos et al [23]	2025	Brazil	Multidomain				✓	ML GRADE (URSE)
Wang et al [58]	2025	China	Imaging				✓	LLM evaluation
Hsu et al [59]	2025	Taiwan	Pharma		✓			LLM extraction
Boyle et al [60]	2025	Canada	Cross-domain	✓	✓			LLM evaluation
Wang et al [61]	2025	China	Drug Monitor				✓	LLM evaluation
von Dincklage et al [62]	2025	Germany	Intensive Care			✓	✓	FHIR (CPG-on-EBMonFHIR)

^a^GEM: guideline elements model.

^b^CIG: computer-interpretable guideline.

^c^GLIF3: Guideline Interchange Format version 3.

^d^GLIA: Guideline Implementability Appraisal.

^e^ML: machine learning.

^f^NLP: natural language processing.

^g^LASSIE: modeling treatment processes using information extraction.

^h^EMR: electronic medical record.

^i^KDOM: Knowledge-Data Ontological Mapper.

^j^DELT/A: Document Exploration and Linking Tool/Addons.

^k^ICU: intensive care unit.

^l^C2PO: Core Clinical Protocol Ontology.

^m^IE: information extraction.

^n^UMLS: Unified Medical Language System.

^o^GRADE: Grading of Recommendations Assessment, Development and Evaluation.

^p^NLU: natural language understanding.

^q^STI: sexually transmitted infection.

^r^FHIR: Fast Healthcare Interoperability Resources.

^s^WHO: World Health Organization.

^t^DAK: Digital Adaptation Kit.

^u^EBM: evidence-based medicine.

^v^ADHD: attention-deficit/hyperactivity disorder.

^w^LLM: large language model.

^x^AGREE II: Appraisal of Guidelines for Research and Evaluation II.

^y^RIGHT: Reporting Items for Practice Guidelines in Healthcare.

**Table 3:** Expanded performance metrics by automation task.

Task	Study	Approach	Performance metrics
Literature retrieval	Bui et al [17]	Query expansion	Recall 80%
Literature retrieval	Fiszman et al [37]	Semantic (SemRep)	Precision 88%; Recall 40%; Specificity 98%
Literature retrieval	Lin et al [55]	Deep learning (PAJO^a^ [PubMedBERT-based)	AUC^b^ 91.84%; *F*_1_-score +1.91% vs baseline; dataset: 89,940 PubMed articles (neck pain)
Literature retrieval	Hu et al [48]	Topic-centric semantic-distance retrieval	Goal evidence identified for 12/16 guideline statements vs 5/16 with non–topic-centric retrieval; evidence-item coverage 46% vs 57%
Info extraction	Kaiser et al [18]	NLP^c^ (LASSIE^d^)	Recall 0.84; Precision 0.87 (treatment steps)
Info extraction	Zhu et al [19]	Rule-based NLP	F-measure: 81.96% (actions), 71.75% (patient states)
Info extraction	Weng et al [46]	Hybrid ontology	86.2% coverage of eligibility criteria
Info extraction	Hsu et al [59]	LLM^e^ (GPT-4o; Gemini-1.5-Pro)	Accuracy 93.5% / 92.7%; Cohen κ 0.90/0.89 (385 PharmGKB annotations, 4-class)
Info extraction	Šuster et al [25]	Transformer (EvidenceGRADEr)	*F*_1_-score 0.74 (overall); *F*_1_-score 0.78 (RoB^f^); *F*_1_-score 0.31 (inconsistency)
Info extraction	Serban et al [35]	Ontology-driven linguistic pattern extraction (Asbru and MHB^g^)	Recall 72%-80%; Precision 20–35%; breast cancer guideline (Asbru and MHB formalization)
Info extraction	Wenzina and Kaiser [63]	Heuristic IE^h^ + UMLS^i^ semantic patterns	Recall 75%; Precision 88%; NPV^j^ 85%; IF-patterns: Recall 82%, Precision 93%
Encoding	Hagerty et al [33]	Semiautomated	Precision 72%-79% (initial heuristics)
Encoding	Georg et al [29]	GEM^k^ vs Manual	Broader coverage (104 vs 98 rules); 37% identical logic
Encoding	González-Ferrer et al [49]	PROForma	Agreement with experts: Cohen κ=0.86 (overall), Cohen κ=0.80 (top diagnosis)
Verification	Bouaud et al [22]	Formal logic	Completeness: 12.6% theoretical coverage; 55% patient coverage
Evaluation	Stewart et al [24]	Bayesian (SAQAT^l^)	Agreement with experts: Cohen κ=0.69
Evaluation	Dos Santos et al [23]	ML (URSE) for GRADE^m^	Accuracy: 97% (imprecision), 90% (heterogeneity), 73% (RoB)
Evaluation	Wan et al [26]	LLM (GPT-4o) for AGREE II^n^ and RIGHT^o^	Domain gaps +22 to +60 pp (LLM > manual, all 6 domains); no ICC^p^ or Cohen κ reported
Evaluation	Wang et al [61]	LLM (GPT-4o) for AGREE II	ICC 0.753 (95% CI 0.532-0.854); mean difference 12.5%, LLM higher (LoA^q^ -30.6% to 55.5%)
GRADE evaluation	López et al [57]	Rule-based GRADE automation (APPRAISE-RS)	Avg 1.96 recommendations per patient (28 simulated); clinician satisfaction median 2.0/3.0; 348 RCTs^r^ (ADHD^s^)

^a^PAJO: Paper title, Abstract, and Journal.

^b^AUC: area under the receiver operating characteristic curve.

^c^NLP: natural language processing.

^d^LASSIE: modeling treatment processes using information extraction.

^e^LLM: large language model.

^f^RoB: risk of bias.

^g^MHB: Many-Headed Bridge.

^h^IE: information extraction.

^i^UMLS: Unified Medical Language System.

^j^NPV: negative predictive value.

^k^GEM: guideline elements model.

^l^SAQAT: Semi-Automated Quality Assessment Tool.

^m^GRADE: Grading of Recommendations Assessment, Development and Evaluation

^n^AGREE II: Appraisal of Guidelines for Research and Evaluation II.

^o^RIGHT: Reporting Items for Practice Guidelines in Healthcare.

^p^ICC: intraclass correlation coefficient.

^q^LoA: limits of agreement.

^r^RCT: randomized controlled trial.

^s^ADHD: attention-deficit/hyperactivity disorder.

**Figure 1:**
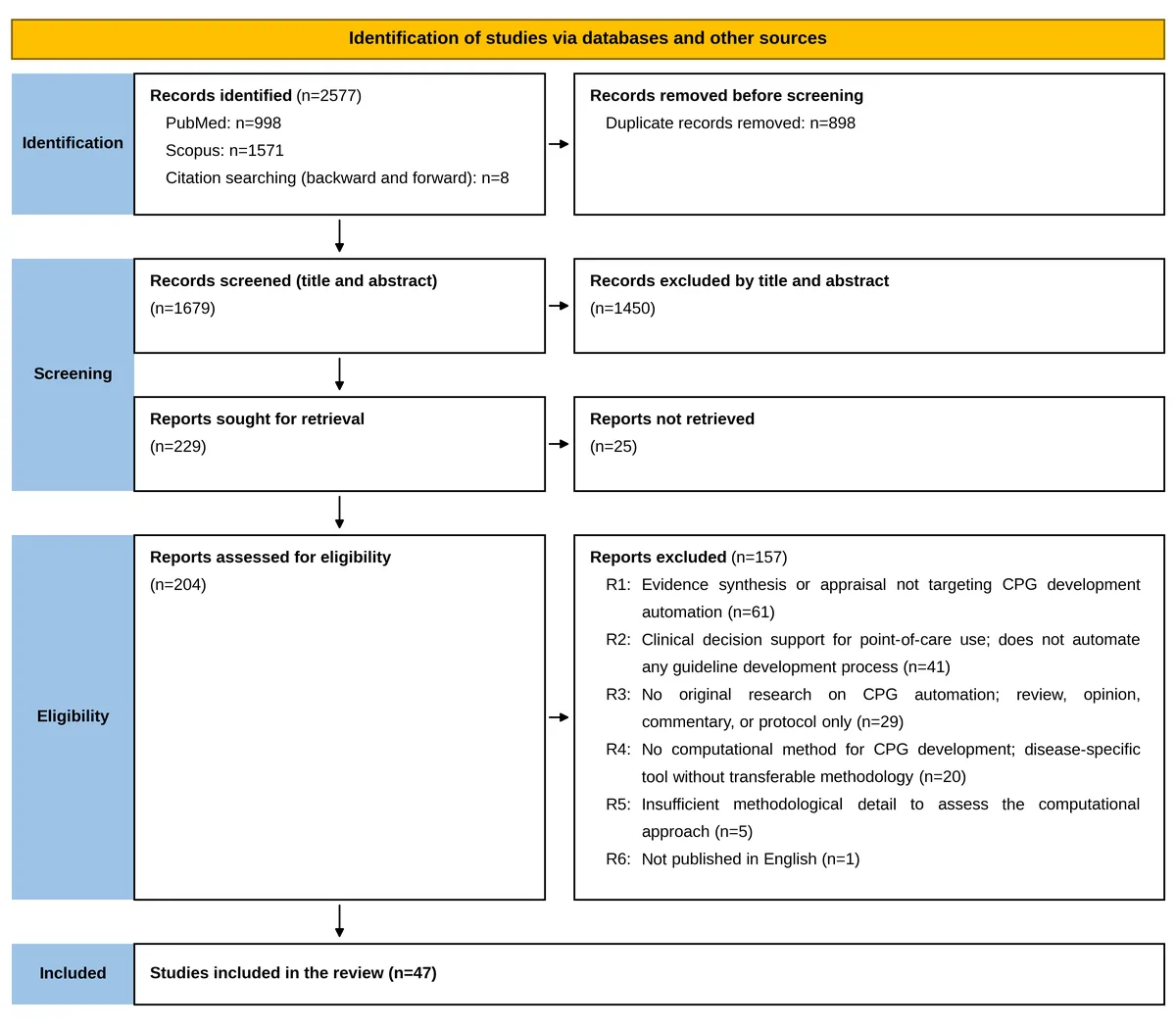
Study selection flow diagram presented using the PRISMA 2020 flow diagram template as adapted for scoping reviews (PRISMA-ScR). Adapted from Page et al [64], which is published under the Creative Commons Attribution 4.0 International License. CPG: clinical practice guideline; R: reason for exclusion.

### Pillar 1: Retrieval

Six studies investigated the automation of literature search and screening, revealing a trade-off between efficiency and comprehensive recall [17,37,48,54,55,60]. Query expansion methods improved literature retrieval, achieving 80% recall in identifying relevant citations [17]. Deep learning approaches demonstrated additional promise: Lin et al [55] proposed the Paper title, Abstract, and Journal (PAJO) model, combining PubMedBERT with journal-level features for automated literature screening in CPG development, achieving an area under the receiver operating characteristic curve (AUC) of 91.84% on a neck pain evidence corpus and outperforming text classification baselines by 1.91% in *F*_1_-score.

However, the transition to semantic and generative search tools highlighted limitations in reliability. While semantic processing tools (eg, SemRep [US National Library of Medicine]) achieved high specificity (98%) and precision (88%) in answering clinical questions, they suffered from low recall (40%), rendering them more suitable for filtering than for the comprehensive searching required in systematic reviews [37]. Furthermore, recent evaluations of generative AI indicated that while LLMs can successfully generate search syntax, they currently fail to independently execute retrieval or screening with the rigorous reliability necessary for systematic reviews [60].

### Pillar 2: Extraction

Fifteen studies addressed the extraction of structured data from narrative text, showing a clear performance divide between rule-based systems and generative models [17-19,25,33,35,37,38,43,46,51,54,59,60,63]. Previously, ontology-driven approaches applied linguistic pattern extraction to map breast cancer guideline text to formal representations (Asbru/MHB [Many-Headed Bridge]), achieving recall of 72%-80% and demonstrating the feasibility of semiautomated formalization from narrative clinical text [35].

Early rule-based NLP systems demonstrated high reliability in specific domains; systems using Unstructured Information Management Architecture (UIMA) and MetaMap (US National Library of Medicine) achieved an F-measure of 81.96% for extracting clinical actions and >70% for fine-grained attributes such as dosage [19]. Hybrid approaches combining NLP with upper-level ontologies (eg, EliXR) successfully covered 86.2% of eligibility criteria in free-text guidelines [46].

In contrast, modern LLM approaches yielded mixed results. While capable of producing structurally coherent recommendations, LLMs demonstrated an accuracy gap between overall performance (77% accuracy) and complex evaluation items (29%-40% accuracy) [58]. Critical evaluations emphasized that LLMs struggle with extraction accuracy compared to human performance, necessitating human-in-the-loop verification to prevent data hallucinations [60].

### Pillar 3: Knowledge Encoding

#### Overview

Twenty-six studies addressed the translation of narrative guidelines into computable formats, highlighting the progression from static formalisms to dynamic, living frameworks [20-22,27-34,36,38-44,49,51-53,56,62,63].

#### Formalisms and Standards

The foundational work in this pillar established flow-chart-based representations and rule-based models. Standards such as GLIF3 (GLIF version 3) were developed to enable shareable, nested, and iterative guideline representations [30], while the GEM was shown to produce rule bases with broader coverage of clinical situations than manually created alternatives [29].

The evolution of encoding standards was accompanied by advances in interoperability and integration. Peleg et al [27] systematically compared 6 major CIG formalisms (Asbru, EON, GLIF, GUIDE, PRODIGY [Prescribing Rationally with Decision Support in General Practice Study], and PROforma) using real clinical guidelines, identifying key modeling differences and paving the way toward consensus standards. Building on this foundation, the same group developed KDOM (Knowledge-Data Ontological Mapper), a framework that automatically generates SQL queries to map GLIF3-encoded guideline data items to institutional electronic medical record (EMR) schemas, enabling cross-institutional sharing of CIGs without manual reencoding [36].

More recently, Fast Healthcare Interoperability Resources (FHIR)–based encoding approaches have extended this interoperability to internationally curated resources. Shivers et al [53] demonstrated automated generation of FHIR Implementation Guides from World Health Organization (WHO) Digital Adaptation Kit data dictionaries for family planning and sexually transmitted infection (STI) guidelines, mapping clinical concepts to standard terminologies (LOINC [Logical Observation Identifiers Names and Codes], SNOMED-CT [Systematized Nomenclature of Medicine Clinical Terms], *ICD-10* [*International Statistical Classification of Diseases, Tenth Revision*], and RxNorm). Further advancing this line of work, an implementation guide directly linking structured evidence artifacts from evidence-based guideline development to computable FHIR CPG recommendations was validated with the COVID-19 dexamethasone recommendation, covering Population, Intervention, Comparison, and Outcome (PICO) relationships, metadata, and GRADE evidence-to-decision justification [56].

#### Semiautomation and Efficiency

Technological interventions have measurably impacted the efficiency of the encoding process. Semiautomated authoring tools have been shown to predict condition and recommendation terms with 72%-79% precision [33]. To address the “living” guideline challenge, integrated systems combining extraction tools with versioning systems (eg, DELT/A [Document Exploration and Linking Tool/Addons]; Vienna University of Technology) allowed for modular updates. These frameworks enabled new guideline versions to inherit unchanged logic from previous iterations, requiring reencoding only for modified text sections [38].

#### Logic and Verification

A distinct advantage of computational encoding identified across multiple studies is the capacity for automated logic verification. Formal encoding methods revealed significant coverage gaps in narrative text, with one study finding that only 12.6% of theoretical patient profiles were explicitly covered by traditional guidelines [22]. Computational logic verification successfully identified anomalies often missed by human reviewers, including contradictory recommendations, dead-end paths [21,28], and temporal conflicts such as overlapping medication schedules [32].

Different formalisms demonstrated specific utility based on the use case: ontologies were found superior for knowledge sharing, while production rules (eg, Drools) excelled at execution speed [20]. In complex clinical scenarios such as hyponatremia, the PROForma formalism achieved high agreement (Cohen κ 0.86) with expert decisions [49], while multilayered models were proposed to separate core guidelines from local adaptations to simplify the update process [52]. Farkash et al [42] used model-driven engineering with Health Level Seven (HL7) standards to produce semiautomated representations readable by physicians.

### Pillar 4: Automated Evaluation

Seventeen studies explored automated quality assessment [23-26,31,39,43,45,47,48,50,54,57,58,61-63].

#### LLM-Based Evaluation

The application of LLMs to the AGREE II instrument was a dominant theme in the 2024-2025 literature. Both studies that compared LLM and human AGREE II appraisal found the same qualitative pattern: the LLM assigned higher absolute scores than human appraisers. The magnitude differed markedly and the two are not on a common scale. Wang et al [61] reported a mean difference of 12.5% across 28 therapeutic drug monitoring guidelines, with 95% limits of agreement of –30.6% to 55.5% and an intraclass correlation coefficient of 0.753. Wan et al [26] report domain-level gaps of 22 to 60 percentage points across all 6 AGREE II domains in 54 documents on day surgery, with no intraclass correlation coefficient or Cohen κ reported. This bias was particularly evident in domains such as “Stakeholder Involvement,” where models tended to hallucinate compliance when details were missing. Despite these accuracy issues, the efficiency gains were substantial, reducing appraisal time from 1.5 hours to under 3 minutes per guideline [61]. Furthermore, when applied to reporting checklists (RIGHT [Reporting Items for Practice Guidelines in Healthcare]), LLMs scored significantly higher than humans (gap ~25%), suggesting a sensitivity to form over substance [26].

#### Consistency and GRADE

Beyond generative AI, formal and machine learning methods demonstrated high utility for specific evaluation tasks. Ontological modeling was successfully used to map patient profiles across different guidelines, confirming logical consistency despite textual variations [45].

For evidence grading, transformer classifiers (EvidenceGRADEr) achieved an overall *F*_1_-score of 0.74, with 0.78 for risk of bias but only 0.31 for inconsistency [25]. A separate machine learning tool for GRADE rating reached 97% accuracy for imprecision, 90% for heterogeneity, and 73% for risk of bias [23]. Performance therefore varies markedly by domain and the two tools are not directly comparable. Bayesian networks provided a structured alternative, achieving substantial agreement (Cohen κ=0.69) with expert judgment, thereby offering a replicable method for quality assessment [24].

Complementing these approaches, APPRAISE-RS is a methodology that automatically applies iterative GRADE criteria to a database of 348 randomized controlled trials (RCTs), generating personalized treatment recommendations. This system achieved good clinician satisfaction in 28 simulated patients and demonstrated the feasibility of automated, continuously updated evaluation at scale [57].

## Discussion

### Summary of Evidence

This scoping review identified 47 studies addressing the automation of clinical practice guideline development, spanning 2001-2025 across 4 pillars [17-63]. In the retrieval pillar (n=6) [17,37,48,54,55,60], the transition from keyword-based filtering to transformer-based classifiers represents the most consequential methodological shift, with deep learning approaches achieving AUC values exceeding 90% on structured evidence corpora [55]. In the extraction pillar (n=15) [17-19,25,33,35,37,38,43,46,51,54,59,60,63], ontology-driven linguistic pattern systems established the baseline [35], while more recent NLP pipelines have extended coverage to complex conditional and temporal constructs; however, recall rates for free-text guideline sentences indicate that human oversight remains necessary [59,60]. In the encoding pillar (n=26) [20-22,27-34,36,38-44,49,51-53,56,62,63], 2 decades of fragmented formalism development have been followed by recent work oriented toward FHIR-based representation. Two studies in this corpus [56,62] demonstrate FHIR implementation guides that produce interoperable, incrementally updatable guideline artifacts compatible with existing health information systems. Whether this represents convergence across the field or an emerging direction cannot be determined from 2 studies [56,62] published within 2 years of each other. In the evaluation pillar (n=17) [23-26,31,39,43,45,47,48,50,54,57,58,61-63], LLM-based tools produced structurally complete AGREE II and GRADE appraisals but assigned higher absolute scores than human appraisers in both studies that made the comparison, with wide limits of agreement [26,61]. This supports their use for triage and prioritization rather than as standalone quality arbiters. Across all pillars, no study has yet demonstrated a fully integrated, validated end-to-end pipeline connecting automated retrieval, extraction, encoding, and evaluation in a production clinical environment. This fragmentation represents the defining knowledge gap of the field.

### Principal Results

A caution applies to every performance figure reported in this review. The included studies used noncommensurable outcome measures—precision, recall, AUC, F-measure, accuracy, Cohen κ, intraclass correlation coefficient, logical coverage, time saved, and clinician satisfaction—assessed against different reference standards, on corpora ranging from 28 guidelines to 89,940 abstracts, and under evaluation conditions ranging from retrospective benchmarking to simulated cases. These values cannot be pooled, ranked, or read as a common scale of maturity. It is more informative to distinguish the dimensions on which evidence exists from those on which it does not. Across the corpus, technical feasibility is well established in all 4 pillars and accuracy in controlled settings is documented for many individual tasks. Reproducibility is reported in only a small number of studies. Interoperability is addressed by the recent FHIR work [56,62]. Clinical safety, workflow integration, scalability, cost-effectiveness, and real-world adoption are essentially unevidenced: no included study reported an evaluation of an automation component embedded in an operating guideline program. The most defensible reading of this literature is therefore that automation demonstrates clear promise for bounded, structured tasks within guideline development, while robust evidence for end-to-end, production-scale living guideline pipelines remains limited.

This scoping review establishes that automated technologies for guideline development have progressed from theoretical proof-of-concept to practical, augmentative tools. A clear dichotomy exists in the current state of the art: rule-based and ontology-driven methods offer the rigorous reliability required for clinical safety and logic verification[20,21,49], while emerging LLMs offer the processing speed needed to handle vast literature volumes [59,61]. However, the latter is currently hindered by “hallucinations” and systematic scoring biases [60,61]. Consequently, a fully automated pipeline remains elusive, with the most successful models using automation for data-intensive retrieval and initial encoding [17,33], while retaining human oversight for contextual interpretation [60,65].

### Comparison With Prior Work

#### The Evolution of Encoding

Encoding represents the highest-leverage investment in the guideline pipeline because it uniquely enables quality gains that narrative guidelines cannot deliver [32,33]. The progression from early formalisms such as GLIF3 and GEM to modern hybrid ontology approaches highlights a shift toward interoperability [29,30]. Crucially, the literature confirms the feasibility of “living” guidelines through modular updates. Systems that allow new guideline versions to inherit unchanged logic from previous versions address the core challenge of keeping pace with daily evidence publication [38,52], a capability that purely narrative updating methods lack.

The inclusion of recent FHIR-based studies strengthens this trajectory considerably. Recent work demonstrated that structured evidence artifacts from the guideline development workflow can now be directly translated into computable FHIR recommendations using WHO Digital Adaptation Kit data dictionaries, mapping clinical concepts to standard terminologies (LOINC, SNOMED-CT, *ICD-10*, and RxNorm) [53]. Building on this, an implementation guide directly linking evidence artifacts from guideline development to computable FHIR CPG recommendations was validated against a real clinical scenario—the COVID-19 dexamethasone recommendation—covering PICO relationships, metadata, and GRADE evidence-to-decision justification [56]. This represents a meaningful step beyond earlier encoding frameworks: where GLIF3 and GEM required significant manual knowledge engineering, modern FHIR-based approaches leverage internationally curated data structures to reduce that burden. Equally, the KDOM framework established the foundational principle of automatic schema mapping between CIG formalisms and institutional EMR data models—a precondition for any truly deployable living guideline system [36]. Together, these studies suggest the field is converging toward a common interoperability layer that was largely absent from earlier reviews.

#### The Role of Generative AI

The integration of recent findings positions LLMs as assistants rather than replacements. A comparison of retrieval and evaluation studies reveals a critical safety limitation: LLMs exhibit reliable structural outputs but struggle with independent factual recall and objective evaluation [59,60]. Both studies that compared LLM and human AGREE II appraisal found the LLM assigned higher scores on average, though the magnitude differed markedly (mean difference 12.5%, 95% limits of agreement –30.6% to 55.5%, in 28 therapeutic drug monitoring guidelines [61]; 22 to 60 percentage points across domains in 54 documents on day surgery [26]). Because the direction was not consistent across guidelines of differing quality [61], these findings support the use of LLMs for summarization and triage rather than for definitive quality assurance. The consensus across the reviewed studies indicates that Generative AI must be constrained by formal verification methods (logic checks) and human review to be safely deployed in clinical guideline development [26,60].

#### Integration of Pillars

The four pillars—retrieval, extraction, encoding, and evaluation—are functionally interdependent. Extraction provides raw material for encoding [18,46]; encoding facilitates automated appraisal of logical consistency [22,28]; and appraisal determines which evidence warrants extraction [15,17]. However, the literature remains fragmented, with few studies bridging these pillars. The path forward identified by this review is the development of end-to-end pipelines where extracted text is automatically mapped to standards such as HL7 FHIR, then verified by logic engines [42].

One study in this corpus provides early empirical evidence that this loop is achievable: starting from a database of 348 RCTs, APPRAISE-RS automatically applied iterative GRADE methodology to generate personalized treatment recommendations for individual patients [57]. While limited to a single disease area (attention-deficit/hyperactivity disorder) and evaluated on simulated cases, it illustrates what a fully automated living guideline pipeline could look like in practice.

### Practical Implications for Digital Health

The findings of this review have direct implications for digital health developers, guideline bodies, and health informaticians. In the retrieval and extraction pillars, transformer-based classifiers and rule-based NLP tools are sufficiently mature for use as screening and extraction assistants in literature surveillance workflows, provided human oversight is retained for borderline cases and interpretive content [19,46,55].

In the encoding pillar, FHIR-based authoring tools—specifically the WHO DAK-to-FHIR pipeline, developed by Shivers et al [53] in collaboration with the WHO, and CPG-on-EBM-on-FHIR, developed by Lichtner et al [56] as part of the COVID-19 Evidence Ecosystem (CEOsys) project—are the outputs of this research program closest to practical deployment. They offer standardized, interoperable artifacts that can integrate with existing health information systems and support incremental updates. Neither has yet been evaluated in routine guideline production.

In the evaluation pillar, LLM-based tools show promise for ranking and flagging guideline quality but should not yet be used as stand-alone assessors given the documented upward scoring bias [26,61]; hybrid workflows combining LLM screening with human confirmation represent the most defensible current approach.

The most significant knowledge gap identified by this review is the absence of validated end-to-end systems connecting all 4 pillars: no existing study has demonstrated a complete pipeline from automated literature retrieval through extraction, encoding, and evaluation to recommendation generation at scale. This represents the primary development priority for the field.

### Limitations

Several features of the search constrain what this review can claim. The search covered 2 databases: PubMed and Scopus. Scopus was included to reach the informatics and engineering literature that a biomedical database indexes incompletely. Even so, it is likely that IEEE Xplore, the ACM Digital Library, and Web of Science would surface further work, particularly in conference proceedings. The search strategy is a building-block design that pairs guideline terms with named formalisms, tasks, and instruments; it favors precision over recall and may underretrieve studies describing comparable methods in vocabulary outside the enumerated pairings, and it was not formally peer-reviewed. Gray literature, preprints, and dissertations were not searched. Eligibility was restricted to English-language publications; one otherwise eligible study was excluded on this basis, and the restriction may underrepresent work published in other languages, particularly from European, Latin American, and East Asian informatics groups active in this field. The search window opens in January 2001, and earlier work is therefore not represented.

The synthesis of evidence was significantly challenged by the substantial study heterogeneity across the included literature. This variability encompassed differences in study design, target clinical domains, specific automation technologies evaluated, and reported outcomes, which collectively precluded the execution of a formal meta-analysis. Consequently, the findings presented are primarily based on a narrative and descriptive synthesis.

Furthermore, it is essential to contextualize the findings within the rapidly evolving landscape of the field, particularly concerning advancements in sophisticated language models such as LLMs and other forms of generative AI [4,61]. Given the pace of innovation, some of the specific technological findings, or the general technological state described in the older included literature (eg, pre-2015 NLP methods), may be potentially dated. Future research will need to continuously update this evidence base to remain current with emerging technologies.

A significant limitation observed across the corpus was that most studies were proof-of-concept demonstrations or early-stage feasibility assessments (Table 2). They often focused on showing what was technologically possible in a controlled environment. As a result, there was a limited body of evidence pertaining to real-world implementation, practical utility, and sustained impact. Crucially, evidence on long-term effectiveness, scalability, cost-effectiveness, and integration into existing clinical workflows remains scarce. The lack of robust implementation evidence makes it difficult to draw definitive conclusions about the true clinical and operational value of these automation solutions.

Finally, the potential for publication bias must be acknowledged. Research dissemination practices may inherently favor the reporting of studies that demonstrate positive or novel findings. This bias could lead to an overrepresentation of successful proof-of-concept evaluations, potentially skewing the perception of efficacy and feasibility, while studies with neutral or negative results, or those focused purely on implementation challenges, may be underrepresented in the published literature.

### Conclusions

Automation of clinical practice guideline development has established technical feasibility across retrieval, extraction, encoding, and evaluation, with the strongest evidence for bounded, well-structured tasks assessed under controlled conditions. Evidence for end-to-end, production-scale living guideline pipelines remains limited: no study in this corpus demonstrated a validated pipeline connecting all 4 pillars in an operating guideline program, and evidence on clinical safety, workflow integration, and cost-effectiveness is largely absent. The pattern across the literature supports a hybrid, human-in-the-loop model in which automation handles data-intensive retrieval and initial encoding while experts provide contextual judgment and validation. Realizing continuously updated living guidelines will require both interoperability standards that connect extraction to computable representation, and evaluation of these components in real guideline production rather than in retrospective benchmarks.

## Appendix

Multimedia Appendix 1 Completed PRISMA-ScR (Preferred Reporting Items for Systematic Reviews and Meta-Analyses extension for Scoping Reviews) checklist and search strategies for PubMed/MEDLINE and Scopus.

## Abbreviations

AUC: area under the receiver operating characteristic curve
AGREE II: Appraisal of Guidelines for Research and Evaluation II
CEOsys: COVID-19 Evidence Ecosystem
CIG: computer-interpretable guideline
CPG: clinical practice guideline
DELT/A: Document Exploration and Linking Tool/Addons
EMR: electronic medical record
FHIR: Fast Healthcare Interoperability Resources
GEM: guideline elements model
GLIA: Guideline Implementability Appraisal
GLIF: Guideline Interchange Format
GLIF3: Guideline Interchange Format version 3
GRADE: Grading of Recommendations Assessment, Development and Evaluation
HL7: Health Level Seven
ICD-10: International Statistical Classification of Diseases, Tenth Revision
JBI: Joanna Briggs Institute
KDOM: Knowledge-Data Ontological Mapper
LLM: large language model
LOINC: Logical Observation Identifiers Names and Codes
MHB: Many-Headed Bridge
NLP: natural language processing
PAJO: Paper title, Abstract, and Journal
PICO: Population, Intervention, Comparison, and Outcome
PRISMA-ScR: Preferred Reporting Items for Systematic Reviews and Meta-Analyses extension for Scoping Reviews
PRODIGY: Prescribing Rationally with Decision Support in General Practice Study
RCT: randomized controlled trial
RIGHT: Reporting Items for Practice Guidelines in Healthcare
SNOMED-CT: Systematized Nomenclature of Medicine Clinical Terms
STI: sexually transmitted infection
UIMA: Unstructured Information Management Architecture.
WHO: World Health Organization

## Data Availability

The datasets generated or analyzed during this study (the data extraction sheet, study-level pillar classifications, and summary tables) are available in the Open Science Framework repository [11].
